# The relationship between Gensini score and rates of 30-day mortality in acute coronary syndrome patients in China

**DOI:** 10.1186/s12872-025-05303-5

**Published:** 2025-11-29

**Authors:** Yi Zhang, Rong Luo, Xiaochen Sun, Jiasheng Cai, Mohammed Ahmed Al-Kaif, Zhonghua Wang, Chunlei Li, Chuntao Wu, Zilong Wang, Zhuqin Li, Haibo Liu

**Affiliations:** 1 Departments of Cardiology, QingPu Branch of Zhongshan Hospital Affiliated to Fudan University, 1158 Park East Road, Shanghai, 201799 China; 2 Department of Cardiology, Huadong Hospiatal affiliated to Fudan University, 221 Yanan West Road, Shanghai, 200040 China; 3 Department of Cardiology, the First Affiliated Hospital of Harbin Medical University, 23 Youzheng Street, Harbin, 150001 China

**Keywords:** Gensini score, Acute coronary syndrome, Mortality, Coronary artery disease, Clinical outcomes

## Abstract

**Background:**

Few studies validated the association between Gensini scores and mortality among acute coronary syndrome (ACS) patients. Therefore, we intend to evaluate the association between the severity of CAD assessed by the Gensini score and the 30-day all-cause mortality in Chinese patients with ACS.

**Methods:**

This study evaluated 2,742 patients diagnosed with ACS who underwent hospitalization and coronary angiography between August 2014 and November 2019 from 2 centers in China. The severity of coronary artery disease (CAD) was categorized into tertiles based on Gensini scores for each lesion: T1 (< 42), T2 (42 ≤ score < 76), and T3 (score ≥ 76). The correlation between Gensini scores and 30-day mortality was assessed using Cox regression analyses and Kaplan-Meier curves.

**Results:**

During the 30-day follow-up period, 2.7% of the patients included in the study occurred mortality. Significant differences were observed in tertile Gensini scores when comparing patients who died (T1: 0.8%; T2: 2.0%; T3: 5.2%, *P* < 0.001). When treating Gensini scores as a continuous variable and adjusting for potential confounding factors, a positive correlation was found between Gensini scores and all-cause mortality (HR 1.012, 95% CI 1.006–1.017). Compared to individuals in the T1 group, those in higher Gensini tertiles exhibited significant increases in all-cause mortality (T2: HR 1.715, 95% CI: 0.704–4.181; T3: HR 2.376, 95% CI: 1.004–5.623; P for trend = 0.039). Subgroup analyses further demonstrated the significant association between higher Gensini scores and increased all-cause mortality was consistently observed across nearly all subgroups.

**Conclusion:**

The present study demonstrated that higher Gensini scores were positively associated with 30-day all-cause mortality among patients with ACS, underscoring their utility as a simple and practical tool for early risk stratification. Further research is warranted to validate these findings and to explore interventions guided by Gensini scores to improve outcomes in this population.

**Supplementary Information:**

The online version contains supplementary material available at 10.1186/s12872-025-05303-5.

## Introduction

Coronary heart disease (CHD) remains a leading cause of death worldwide [1]. Acute coronary syndrome (ACS), including ST-segment elevation myocardial infarction (STEMI) and non-ST-segment elevation ACS (NSTE-ACS), is a critical presentation of CHD that impacts over 7 million individuals annually, which contributes to significant morbidities and mortalities [2]. Although percutaneous coronary intervention (PCI) or fibrinolytic therapy can reduce mortality rates among patients with ACS, a mortality of 4.9–7.0.9.0% persists in this cohort [2]. Thus, there is a pressing need to define clinical risk factors that can aid in identifying risk patients so that therapeutic interventions can be tailored to maximize the odds of a positive outcome [3].

Coronary artery lesion characteristics have been established as a key prognostic factor in coronary artery disease (CAD) patients, leading to the development of various scoring systems for the quantitative evaluation of these lesions, including Gensini scores, SYNTAX scores, American College of Cardiology/American Heart Association (ACC/AHA) scores, and Leaman scores [4–8]. Gensini scoring is an exceptionally robust tool for lesion assessment by integrating lesion numbers, locations, and the associated degree of stenosis [9–12]. And there are no other better tools to measure stenosis and occlusion, which reflect the amount of atherosclerosis process. Limited evidence suggests that Gensini scores are related to cardiovascular and cerebrovascular event incidence among CAD patients [8, 13], but the association between these scores and ACS patient mortality remains to be assessed. In this retrospective cohort study, we sought to investigate the relationship between Gensini scores and 30-day all-cause mortality in patients with ACS in China.

## Methods

### Study design and population

**Fig. 1:**
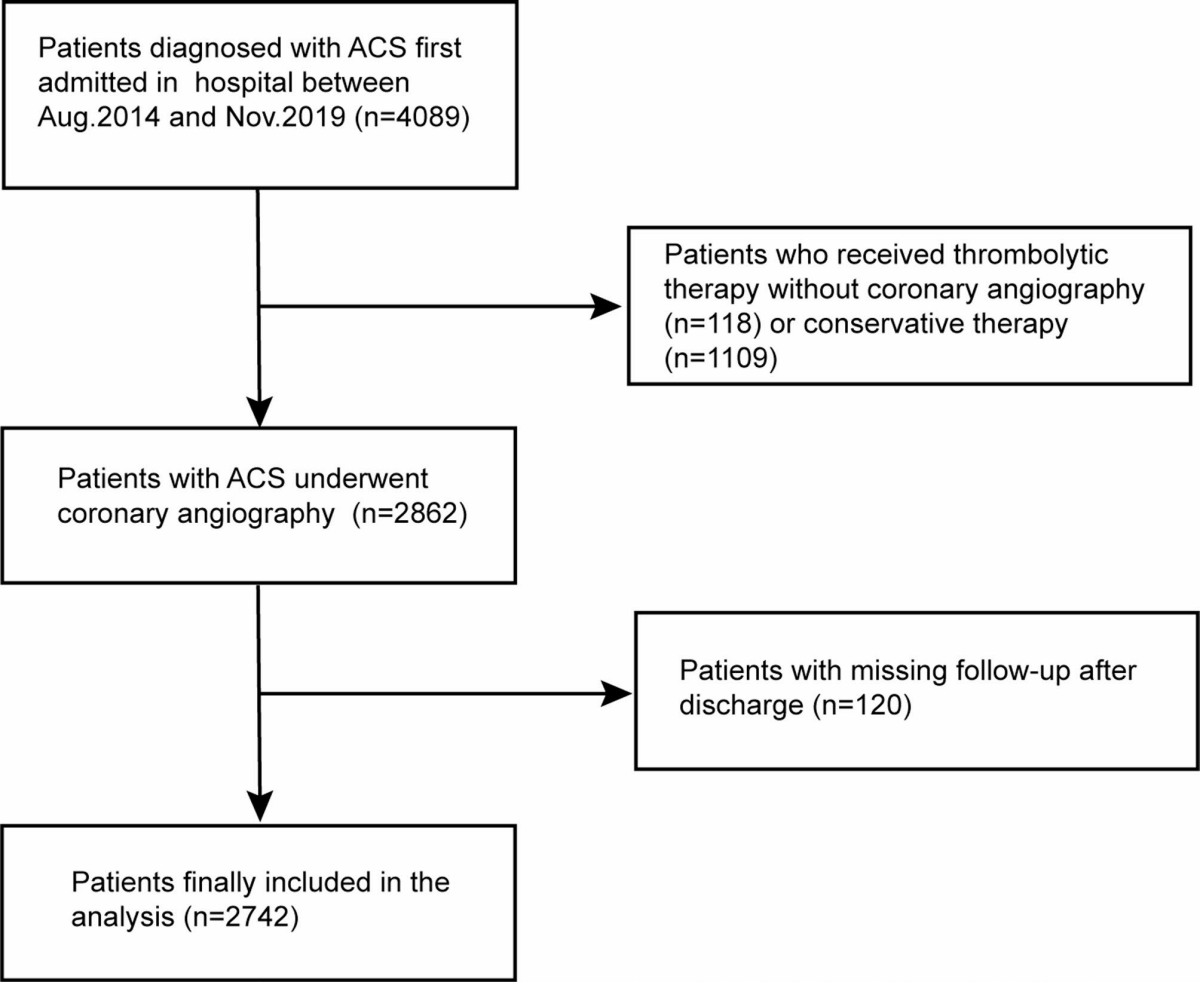
Flowchart of patient selection

Our study was retrospective and included 2,742 patients with ACS who underwent hospitalization and coronary angiography in the cardiovascular department of the First Affiliated Hospital of Harbin Medical University and Qingpu Branch of Zhongshan Hospital Affiliated with Fudan University from August 2014 – November 2019. Data were obtained from case records and follow-up registries. Patients eligible for inclusion were individuals diagnosed with ACS who were first admitted for hospitalization (*n* = 4,089). Patients were excluded if they underwent treatment with thrombolytic therapy alone and did not undergo coronary angiography (*n* = 118), were administered conservative drug therapy (*n* = 1,109), or were missing post-discharge follow-up data (*n* = 120). The remaining 2,742 ACS patients (NSTE-ACS: 1,058; STEMI: 1684) were enrolled in this study (Fig. 1). This study received ethical approval from the ethics committee of the First Affiliated Hospital of Harbin Medical University and Qingpu Branch of Zhongshan Hospital Affiliated with Fudan University and adhered to the principles outlined in the Declaration of Helsinki. Due to the retrospective nature of the analyses conducted, the requirement for informed consent was waived and the clinical trial number was not applicable.

### Patient characterization

The demographic and clinical characteristics of the patients included in this study encompassed various factors. These factors consisted of age, sex, smoking habits, drinking habits, systolic and diastolic blood pressure (SBP and DBP), ACS type, Killip classification, comorbid conditions (such as hypertension, diabetes, previous heart failure [HF], previous myocardial infarction [MI), history of stroke, peripheral artery disease, history of cancer, and valvular heart diseases), and medication use while hospitalized. To assess the hematological and biochemical parameters of the patient’s blood samples obtained for analysis purposes in this study, Beckman Coulter 6800 and Roche Cobas 8000 instruments were utilized. Furthermore, a 2D-guided M-mode echocardiographic approach was employed to evaluate left ventricular ejection fraction (LVEF) upon admission. Angiographic and procedural details were also documented. These details encompassed aspects such as intra-aortic balloon pump (IABP), stent implantation, infarct-related artery, multivessel disease, triple vessel disease [14], and complete coronary revascularization. The concomitant medication included dual antiplatelet, statin, β-blocker, angiotensin-converting enzyme inhibitors or angiotensin II receptor blockers (ACEI or ARB), and low molecular weight heparin (LMWH) or unfractionated heparin (UFH).

The guidelines published by the European Society of Cardiology (ESC) were employed to define heart failure [15] and valvular heart diseases [16]. Diagnosing peripheral artery disease in hospitalized patients relied on clinical history and ankle-brachial index assessment [17]. The definitions of primary PCI and complete coronary revascularization were established in accordance with prior literature [18–20]. 

### Coronary angiography

All patients in the study underwent coronary angiography, and at least two interventional physicians analyzed the resulting angiograms. Two senior cardiologists independently used the Gensini system to assess the severity of CAD before reperfusion therapy. The Gensini scoring system assigns a severity score to each lesion based on its location and degree of stenosis.

The degree of stenosis was estimated as follows: ≤ 25% stenosis, 1 point; 26–50% stenosis, 2 points; 51–75% stenosis, 4 points; 76–90% stenosis, 8 points; 91–99% stenosis, 16 points; total occlusion, 32 points. Additionally, each lesion score was multiplied by a region multiplication factor (the left main coronary artery [LM], 5; the proximal part of the left anterior descending artery [LAD] and the proximal part of the left circumflex artery [LCX], 2.5; the mid-part of the LAD, 1.5; the right coronary artery [RCA], the distal portion of the LAD, the obtuse marginal artery and the posterolateral artery, 1.0; and other parts 0.5) (Supplemental Table 1).

Finally, individual coronary segment scores were summarized to calculate the Gensini score [21]. To facilitate analysis and comparison among patients, they were categorized into three groups based on tertiles of their Gensini scores: T1 (< 42), T2 (42 ≤ score < 76), and T3 (score ≥ 76)^4^.

### Outcomes

The primary study outcome was 30-day all-cause mortality following cardiovascular disease unit admission. Mortality data were obtained through electronic medical record review and phone calls to patients or their families within 2 months following discharge.

### Statistical analyses

Categorical data were reported as frequencies with percentages, whereas continuous data were means ± SD. Gensini scores were evaluated as a continuous variable grouped into tertiles (T1 < 42; 42 ≤ T2 < 76; T3 ≥ 76). ANOVAs and Kruskal-Wallis tests were employed when analyzing continuous data that were not normally distributed, while categorical data were assessed with chi-square tests. Kaplan-Meier curves and log-rank tests were used when evaluating differences in 30-day mortality among Gensini score tertiles. Relationships between Gensini scores and all-cause mortality were assessed through multivariate analyses using hazard ratios (HRs) and corresponding 95% confidence intervals (CIs). Models were first minimally adjusted for patient age, sex, SBP and Killip classification I-II. Models were then fully adjusted for age, sex, SBP, Killip classification I-II, previous MI, previous HF, Troponin I levels (TnI) at admission, N-terminal pro-brain natriuretic peptide (NT-proBNP), high-sensitivity C-reactive protein (Hs-CRP) levels, LVEF, primary PCI, thrombolytic therapy, triple-vessel disease, left main artery (LM) culprit, stent implantation, IABP, complete coronary revascularization, ACEI or ARB, and dual antiplatelet medication. The factors included in the fully adjusted model were selected because they were correlated with 30-day all-cause mortality or with a greater than 10% change in effect estimates [22]. Area under the receiver operating characteristic (ROC) curve (AUC) values were employed when assessing the utility of Genisi scores as predictors of 30-day mortality. Missing values were evident for < 5% of cases for several covariates including Hs-CRP, NT-proBNP, and LVEF, and were accounted for through multiple imputations using chained equations via the R mice 1.16.0 package based on five replicates to minimize statistical effects in the associated analyses [23].

Relationships between Gensini scores and 30-day all-cause mortality were additionally explored by conducting subgroup analyses [4, 24, 25], which were conducted with R 3.4.3 (http://www.R-project.org) and EmpowerStats (http://www.empowerstats.com). *P* < 0.05 served as the statistical threshold used to define significance.

## Results

### Patient baseline characteristics

The patients enrolled in this study were initially grouped into three Gensini score tertiles as follows: T1 (< 42, *n* = 901), T2 (≥ 42, < 76, *n* = 901), and T3 (≥ 76, *n* = 940). Compared with the other tertiles, patients in the T1 group were more likely to be younger, to present with Killip classification I–II, to have NSTE-ACS, to exhibit higher LVEF, and to undergo complete coronary revascularization and stent implantation. In contrast, patients in the T3 group were more often older and had higher heart rate and hs-CRP levels, STEMI, diabetes, prior HF, previous MI, history of stroke, elevated NT-proBNP, IABP, multivessel or triple-vessel disease, primary PCI, and β-blocker treatment. No differences were observed among these groups concerning other variables, including sex, smoking habits, drinking habits, DBP, hypertension, peripheral artery disease, history of cancer, valvular heart diseases, hemoglobin, serum creatine, high-density lipoprotein cholesterol (HDL-C), low-density lipoprotein cholesterol (LDL-C), TnI, creatine kinase myocardial band content (CK-MB), thrombolytic therapy, both of thrombolytic therapy and rescue PCI therapy, coronary artery bypass graft (CABG) surgery, dual antiplatelet, statin, ACEI or ARB, and LMWH or UFH treatment. Table 1

**Table 1:** Baseline characteristics of patients

Variables	Total	Gensini scores			
		T1	T2	T3	*P*-value
		(< 42 points)	(≥ 42, < 76 points)	(≥ 76 points)	
N	2742	901	901	940	
Age, y	59.8 ± 11.0	58.3 ± 11.3	60.2 ± 10.5	60.9 ± 11.0	< 0.001
Male, n (%)	1916 (69.9%)	640 (71.0%)	618 (68.6%)	658 (70.0%)	0.526
Smoking habits, n (%)	1339 (48.8%)	452 (50.2%)	457 (50.7%)	430 (45.7%)	0.063
Drinking habits, n (%)	545 (19.9%)	190 (21.1%)	177 (19.6%)	178 (19.0%)	0.507
Heart rate, beats/min	76.3 ± 16.8	73.8 ± 16.1	75.7 ± 16.0	79.4 ± 17.8	< 0.001
SBP, mmHg	133.2 ± 25.2	131.9 ± 23.9	135.2 ± 25.9	132.7 ± 25.8	0.014
DBP, mmHg	81.8 ± 15.8	80.9 ± 15.6	82.4 ± 15.8	82.2 ± 16.0	0.093
Killip classification I-II, n (%)	2619 (95.5%)	876 (97.2%)	870 (96.6%)	873 (92.9%)	< 0.001
ACS type, n (%)					< 0.001
NSTE-ACS	1058 (38.6%)	378 (42.0%)	365 (40.5%)	315 (33.5%)	
STEMI	1684 (61.4%)	523 (58.0%)	536 (59.5%)	625 (66.5%)	
Comorbidities, *n* (%)					
Hypertension	1398 (51.0%)	440 (48.8%)	458 (50.8%)	500 (53.2%)	0.173
Diabetes	604 (22.0%)	168 (18.6%)	207 (23.0%)	229 (24.4%)	0.009
Previous HF	367 (13.4%)	71 (7.9%)	98 (10.9%)	198 (21.1%)	< 0.001
Previous MI	175 (6.4%)	40 (4.4%)	60 (6.7%)	75 (8.0%)	0.007
History of stroke	379 (13.8%)	103 (11.4%)	134 (14.9%)	142 (15.1%)	0.040
Peripheral artery disease	11 (0.4%)	3 (0.3%)	4 (0.4%)	4 (0.4%)	0.923
History of cancer	12 (0.4%)	6 (0.7%)	3 (0.3%)	3 (0.3%)	0.448
Valvular heart disease	10 (0.4%)	4 (0.4%)	4 (0.4%)	2 (0.2%)	0.635
Hematological, biochemical and echocardiography variables					
WBC, 10^9/L	18.1 ± 4.9	18.2 ± 4.8	18.4 ± 4.9	17.8 ± 4.9	0.036
Hemoglobin, g/L	142.8 ± 18.1	142.5 ± 17.3	142.5 ± 17.6	143.4 ± 19.3	0.509
Hs-CRP, mg/L	19.2 ± 30.4	16.5 ± 26.4	18.9 ± 30.5	22.1 ± 33.4	< 0.001
Serum creatine, umol/L	71.4 ± 27.6	70.6 ± 24.5	72.0 ± 30.4	71.6 ± 27.7	0.553
HDL-C, mmol/L	1.1 ± 0.2	1.1 ± 0.2	1.1 ± 0.2	1.1 ± 0.3	0.763
LDL-C, mmol/L	3.2 ± 0.8	3.1 ± 0.8	3.2 ± 0.8	3.2 ± 0.8	0.175
TnI level at admission, ng/ml	0.9 [0.1, 9.0]	0.9 [0.1–9.0.1.0]	1.1 [0.1–8.6]	0.9 [0.1–9.0.1.0]	0.922
CK-MB, ng/ml	14.4 [3.7, 43.0]	14.0 [4.7, 42.0]	14.0 [3.3, 43.4]	15.0 [3.6, 44.7]	0.669
NT-proBNP, ng/L	1145.6 [598.6–2483.5.6.5]	914.9 [516.8, 1923.0]	1141.4 [579.1, 2242.8]	1458.0 [704.0, 3345.6]	< 0.001
LVEF, %	54.3 ± 9.4	56.6 ± 8.5	55.0 ± 9.0	51.5 ± 9.7	< 0.001
Angiographic and procedural characteristics, *n* (%)					
Stent implantation	2323 (84.7%)	778 (86.3%)	777 (86.2%)	768 (81.7%)	0.007
IABP	9 (0.3%)	0 (0.0%)	0 (0.0%)	9 (1.0%)	< 0.001
Multivessel disease	1768 (64.5%)	384 (42.6%)	649 (72.0%)	735 (78.2%)	< 0.001
Triple vessel disease	757 (27.6%)	82 (9.1%)	229 (25.4%)	446 (47.4%)	< 0.001
Infarct-related artery					
LM	126 (4.6%)	3 (0.3%)	22 (2.4%)	101 (10.7%)	< 0.001
LAD	2143 (78.2%)	486 (53.9%)	776 (86.1%)	881 (93.7%)	< 0.001
LCX	1384 (50.5)	329 (36.5%)	441 (48.9%)	614 (65.3%)	< 0.001
RCA	1706 (62.2)	527 (59.5%)	561 (62.3%)	618 (65.7%)	0.006
Primary PCI	1159 (42.3%)	343 (38.1%)	355 (39.4%)	461 (49.0%)	< 0.001
Thrombolytic therapy	72 (2.6%)	28 (3.1%)	25 (2.8%)	19 (2.0%)	0.321
Both of thrombolytic and rescue PCI therapy	57 (2.1%)	22 (2.4%)	20 (2.2%)	15 (1.6%)	0.414
Complete coronary revascularization	643 (23.5%)	347 (38.5%)	133 (14.8%)	163 (17.3%)	< 0.001
CABG	5 (0.2%)	0 (0.0%)	2 (0.2%)	3 (0.3%)	0.074
Concomitant medication, n (%)					
Dual antiplatelet	2639 (96.2%)	875 (97.1%)	857 (95.1%)	907 (96.5%)	0.074
Statin	2485 (90.6%)	820 (91.0%)	820 (91.0%)	845 (89.9%)	0.636
β-blocker	1663 (60.6%)	495 (54.9%)	560 (62.2%)	608 (64.7%)	< 0.001
ACEI or ARB	656 (23.9%)	204 (22.6%)	219 (24.3%)	233 (24.8%)	0.530
LMWH or UFH	291 (10.6%)	91 (10.1%)	93 (10.3%)	107 (11.4%)	0.635

Mean ± SD for normally distributed continuous variables, median [Q1, Q3] for non-normally distributed continuous variables: *P*-value was calculated by one-way ANOVAs. Number () for categorical variables: *P*-value was calculated by chi-square test

Abbreviations: *ACS* acute coronary syndrome, *ACEI* angiotensin-converting enzyme inhibitor, *ARB* angiotensin receptor blocker, *CABG* coronary artery bypass graft, *CK-MB* creatine kinase myocardial band content, *DBP* diastolic blood pressure, *HDL-C* high-density lipoprotein cholesterol, *HF* heart failure, *Hs-CRP* high-sensitivity C-reactive protein, *IABP* intra-aortic balloon pump, *LAD* left anterior descending artery, *LCX* left circumflex artery, *LVEF* left ventricular ejection fraction, *LM* left main artery, *MI* myocardial infarction, *NSTE-ACS* non-ST-segment elevation acute coronary syndrome, *NT-proBNP* N-terminal pro-brain natriuretic peptide, *PCI* percutaneous coronary intervention, *RCA* right coronary artery, *SBP* systolic blood pressure, *STEMI* ST-segment elevation myocardial infarction, *TnI* Troponin I, *UFH* unfractionated heparin, *WBC* white blood cell

### Relationships between Gensini scores and all-cause mortality

**Fig. 2:**
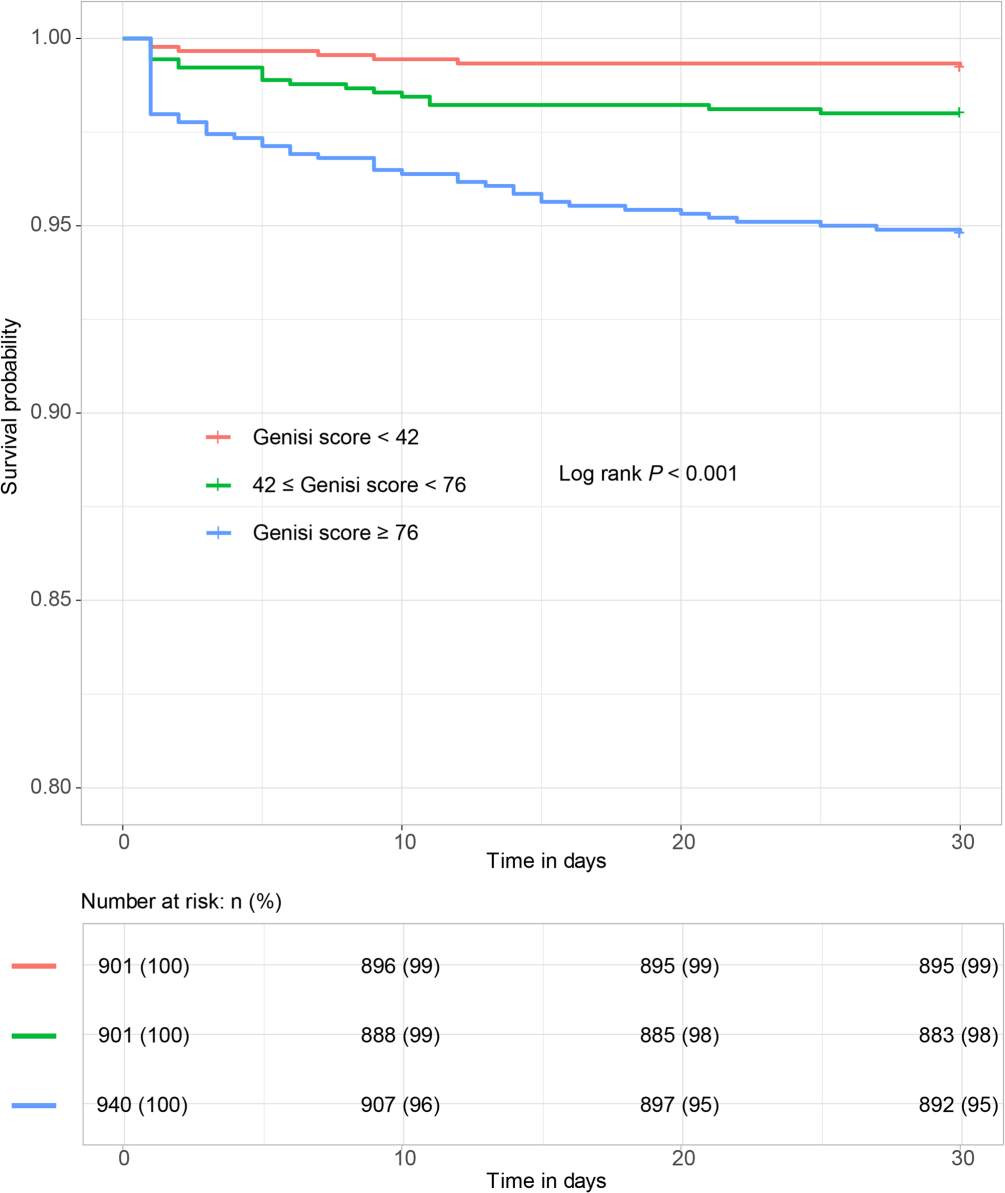
Kaplan–Meier survival curve analysis of 30-day all-cause mortality across the tertiles of the Gensini scores

The overall 30-day all-cause mortality was 2.7% (74 events), with rates of 0.8% in T1, 2.0% in T2, and 5.2% in T3 (Table 2).We then evaluated the associations between Gensini scores and 30-day all-cause mortality were evaluated. When treating Gensini scores as a continuous variable, every unit increase was associated with a corresponding rise in 30-day all-cause mortality risk under the unadjusted model (HR 1.020, 95% CI 1.016, 1.023, *P* < 0.001). This association also remained significant following full adjustment for age, sex, SBP, Killip classification I–II, previous MI, previous HF, TnI, NT-proBNP, Hs-CRP levels, LVEF, primary PCI, thrombolytic therapy, triple-vessel disease, LM culprit, stent implantation, IABP, complete coronary revascularization, ACEI or ARB, and dual antiplatelet medication (HR 1.012, 95% CI 1.006, 1.017, *P* < 0.001). Compared to individuals in the T1 group, higher Gensini score tertiles showed a higher risk of all-cause mortality under in the unadjusted and fully adjusted models (Unadjusted, T2: HR 2.583, 95% CI 1.079, 6.185, T3: HR 6.827, 95% CI 3.092, 15.072, P for trend < 0.001; Fully adjusted, T2: HR 1.715, 95% CI 0.704, 4.181, T3: HR 2.376, 95% CI 1.004, 5.623, P for trend = 0.039). Kaplan-Meier analyses indicated that 30-day all-cause mortality rates rose with increases in Gensini scores, with the best survival outcomes for those ACS in the T1 group (*P* < 0.001) (Fig. 2). Significant AUC values were also evident when exploring the relationships between Gensini scores and 30-day all-cause mortality (AUC 0.755, 95% CI 0.699, 0.813), highlighting the utility of Gensini scores when predicting the adverse clinical outcomes (Supplemental Fig. 1).

**Table 2:** Associations between Gensini scores and all-cause mortality by Cox analysis

	The number of events (deaths), *n* (%)	Unadjusted, HR (95%CI, *P*)	Minimally adjusted, HR (95%CI, *P*)	Fully adjusted, HR (95%CI, *P*)
Gensini scores, points	74 (2.7)	1.020 (1.016, 1.023) < 0.001	1.019 (1.015, 1.023) < 0.001	1.012 (1.006, 1.017) < 0.001
Gensini scores (tertiles)				
T1	7 (0.8)	Ref	Ref	Ref
T2	18 (2.0)	2.583 (1.079, 6.185) 0.033	2.424 (1.012, 5.809) 0.047	1.715 (0.704, 4.181) 0.235
T3	49 (5.2)	6.827 (3.092, 15.072) < 0.001	5.156 (2.319, 11.462) < 0.001	2.376 (1.004, 5.623) 0.049
*P* for trend	--	< 0.001	< 0.001	0.039

Minimally adjusted HR controls for age, sex, SBP, and Killip classification I-II. Fully adjusted HR controls for age, sex, SBP, Killip classification I-II, previous MI, previous HF, TnI, NT-proBNP, Hs-CRP levels, LVEF, primary PCI, thrombolytic therapy, triple-vessel disease, LM culprit, stent implantation, IABP, complete coronary revascularization, ACEI or ARB, and dual antiplatelet medication

Abbreviations: *ACEI* angiotensin-converting enzyme inhibitor, *ARB* angiotensin receptor blocker, *HF* heart failure; *Hs-CRP* high-sensitivity C-reactive protein, *IABP* intra-aortic balloon pump, *LM* left main artery, *LVEF* left ventricular ejection fraction, *MI* myocardial infarction, *NT-proBNP* N-terminal pro-brain natriuretic peptide, *PCI* percutaneous coronary intervention, *SBP* systolic blood pressure, *TnI* Troponin I

### Relationships between Gensini scores and mortality in different subgroups of ACS patients

**Fig. 3:**
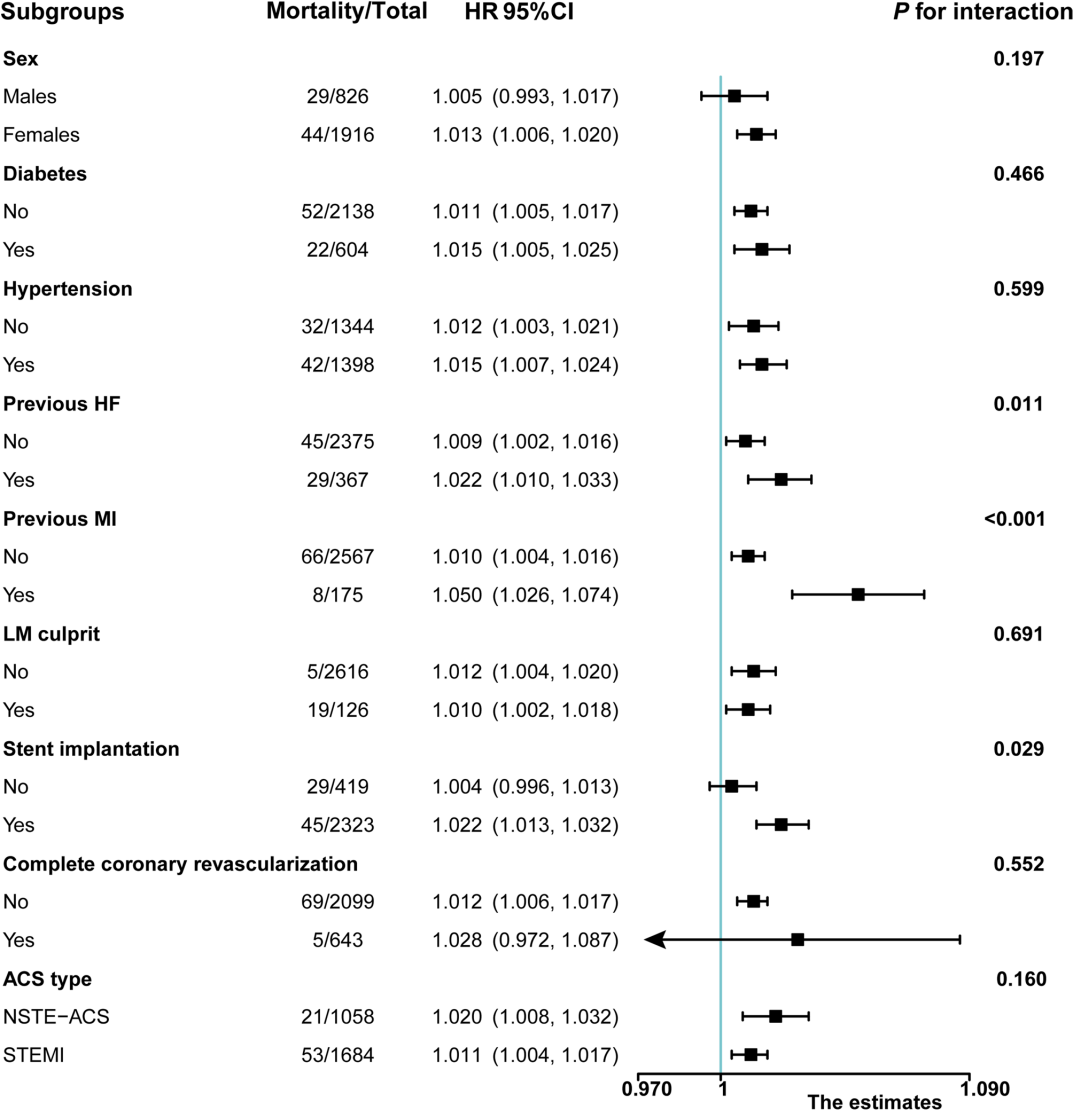
Relationships of the Gensini scores and 30-day all-cause mortality in subgroups. Notes: HRs were adjusted for a age, sex, SBP, and Killip classification. Fully adjusted HR controls for age, sex, SBP, Killip classification I-II, previous MI, previous HF, TnI, NT-proBNP, Hs-CRP levels, LVEF, primary PCI, thrombolytic therapy, triple-vessel disease, LM culprit, stent implantation, IABP, complete coronary revascularization, ACEI or ARB, and dual antiplatelet medication. In each case, the stratification variable itself in each subgroup analysis was not included in the adjusted model. Abbreviations: ACEI: angiotensin-converting enzyme inhibitor; ARB: angiotensin receptor blocker; ACS: acute coronary syndrome; HF: heart failure; Hs-CRP: high-sensitivity C-reactive protein; IABP: intra-aortic balloon pump; LM: left main artery; LVEF: left ventricular ejection fraction; MI: myocardial infarction; NT-proBNP: N-terminal pro-brain natriuretic peptide; NSTE-ACS: non-ST-segment elevation acute coronary syndrome; PCI: percutaneous coronary intervention; SBP: systolic blood pressure; STEMI: ST-segment elevation myocardial infarction; TnI: Troponin I

Higher Gensini scores were positively and significantly correlated with mortality in almost all analyzed ACS patient subgroups (Fig. 3). Specifically, Gensini scores were significantly and positively associated with all-cause mortality in patient subgroups including females, individuals with or without diabetes, individuals with or without hypertension, individuals with or without previous HF, individuals with or without previous MI, individuals with or without LM culprit, individuals with stent implantation, individuals without complete coronary revascularization, and individuals that were primarily admitted for STEMI or NSTE-ACS. Significant interactions were detected between the all-cause mortality endpoint and patients with previous HF, previous MI, and stent implantation (P for interaction < 0.05).

## Discussion

Gensini scores, which are computed based on the locations, number, and stenosis of coronary artery lesions, were herein found to be positively correlated with both 30-day all-cause mortality among patients with ACS in China. Even after controlling for the potential confounding factors, including age, sex, SBP, Killip classification I-II, previous MI, previous HF, TnI, NT-proBNP, Hs-CRP levels, LVEF, primary PCI, thrombolytic therapy, triple-vessel disease, LM culprit, stent implantation, IABP, complete coronary revascularization, ACEI or ARB, and dual antiplatelet medication, a one-unit Gensini score increase was found to be associated with 1.2% increases in all-cause mortality, thus demonstrating an independent and positive correlation between Gensini scores and the clinically relevant endpoints.

Many scoring systems have been designed to to quantitatively assess the extent and severity of coronary artery disease, among which the Gensini and SYNTAX scores are the most widely used, each with distinct strengths and limitations [26]. The SYNTAX score was developed to grade the anatomical complexity of coronary artery disease and to guide revascularization strategies [27]. The integration of multiple angiographic parameters aids in the decision-making process between PCI and CABG [28]. Conversely, Gensini score was developed to quantify the overall burden of coronary atherosclerosis, utilizing only two parameters per lesion: a severity score and a region multiplication factor, thereby simplifying its application in both clinical and research contexts [29]. While the SYNTAX score is primarily utilized as a procedural tool, the Gensini score provides a complementary evaluation of plaque burden and atherosclerotic severity, which may underlie both short- and long-term adverse outcomes. In this context, our study expands the clinical applicability of Gensini score by highlighting its association with short-term outcomes in patients with ACS.

Here, Gensini scores were slightly higher in this ACS patient population than prior research focused on CHD patients [4, 13, 30]. Wang et al. [4] revealed lower cutoff points in patients with CAD who underwent PCI, and Yokokawa et al. [24] reported lower median Gensini scores among heart failure patients (32 and 46 points, respectively, in the low and high residual Gensini score groups). In one cross-sectional analysis of 2,740 new-onset AMI patients, 27.2% exhibited Gensini scores greater than 100 points, with a median Gensini score that was slightly higher than in the present analysis (72.0 vs. 54.3 points) [31]. Qin et al. also found that AMI patients exhibited higher Gensini scores, with median values of 71.5 and 58.0 points in individuals with and without hyperglycemia [32]. As the Gensini scores in the present analysis were in line with those of AMI patients but higher than those for individuals with CHD disease, this may suggest a higher degree of coronary severity among AMI patients. Furthermore, the findings revealed that the prevalence of NSTE-ACS was highest in the T1 group, while conversely, the occurrences of STEMI were highest in the T3 group. The complexity of coronary lesions and the presence of 100% coronary artery stenosis may contribute to a higher severity score in STEMI compared to NSTE-ACS [2]. Aditionally, our analysis indicated that the proportion of patients undergoing primary PCI increased with elevated Gensini scores, which may be associated with the higher incidence of STEMI in patients with increased Gensini scores. Therefore, the ACS subgroup may influence both Gensini score and treatment selection. Nonetheless, the conclusion that Gensini scores exhibit a positive correlation with 30-day all-cause mortality remains consistently robust.

Previous studies have established a correlation between the severity of coronary artery stenosis and long-term adverse cardiovascular events in patients with CHD. Wang et al. [4, 25] revealed that higher Gensini scores were linked to increased long-term all-cause mortality in patients with or without diabetes, with poorer outcomes observed among diabetic patients. In our analysis, although hazard ratios for diabetic patients were numerically higher, no significant interaction was found between diabetes and the association of Gensini scores with 30-day mortality, possibly reflecting undiagnosed diabetes or the overpowering impact of acute events, where overall atherosclerotic burden becomes the predominant determinant of short-term survival. Similarly, Sinning et al. [13] reported that Gensini score effectively predicted long-term outcomes, with its prognostic value further enhanced when integrated with other clinical parameters and comorbidities [33]. Consistent with the findings of Huang et al. [8], who reported an association between Gensini scores and 90-day major adverse cardiovascular events in ACS patients, our study demonstrated that higher Gensini scores were independently associated with 30-day all-cause mortality, supporting the importance of plaque burden even for short-term outcomes.

Given that the Gensini score reflects chronic atherosclerotic burden, an intriguing question arising from our findings is why it is associated with acute, short-term outcomes. We propose that systemic inflammation may represent a key mechanistic link, as several inflammatory indices—including the C-reactive protein–albumin–lymphocyte (CALLY) index, platelet-to-lymphocyte ratio (PLR), CRP, interleukin-1β (IL-1β), and neutrophil-to-lymphocyte ratio (NLR)—have been associated with both Gensini score and short-term adverse events [34–38]. In our cohort, patients in the highest Gensini tertile exhibited elevated high-sensitivity CRP levels, suggesting that inflammation may partly mediate this association. In addition, greater atherosclerotic burden may heighten vulnerability to acute physiological stress, contributing to adverse short-term events. Chronic atherosclerotic changes may impair vascular dynamics, hemodynamics, and myocardial oxygen supply, potentially triggering acute ischemic events [39]. Metabolic stressors such as stress hyperglycemia in ACS are linked to higher Gensini scores and increased incidence of major adverse cardiac events [32], suggesting a synergistic interplay between metabolic dysregulation and atherosclerotic burden. Taken together, these findings indicate that both plaque burden and acute physiological stressors, including metabolic and inflammatory factors, contribute to short-term mortality in ACS patients, underscoring the clinical relevance of the Gensini score beyond long-term risk assessment.

Subgroup analyses confirmed a significant independent association between higher Gensini scores and a greater risk of 30-day mortality in almost all subgroups. Through subgroup and interaction analyses, this link between Gensini scores and all-cause mortality was further found to be influenced by previous HF or MI, and stent implantation, with higher HR values for ACS patients with these comorbid conditions, highlighting an important focus for further research efforts.

The study has several limitations. First, as a single-center, retrospective analysis of patients with ACS, the findings may not be generalizable to other populations and are inherently subject to the biases associated with retrospective study designs. Second, the lack of complete information on specific causes of death limited our ability to analyze cardiovascular mortality separately. Therefore, all-cause mortality was used as the primary endpoint, which may slightly limit the specificity of the findings regarding cardiovascular risk. Third, Gensini scores were assessed before the timing of reperfusion therapy, which may be influenced by the initial TIMI flow. Additionally, on-site angiographic assessments may differ from those adjudicated by a core laboratory, potentially influencing Gensini scores. Fourth, although the Gensini score is a well-established marker for assessing coronary severity in the study, we did not incorporate SYNTAX, ACC/AHA, or Leaman scores for comparative analysis. The absence of these additional scoring systems limits our capacity to contextualize the Gensini scores relative to other established metrics of CAD severity and restricts direct comparisons among these scoring systems [40]. Finnally, in some instances, ascertainment of death was based on telephone follow-up with family members rather than hospital records, which may have introduced a degree of misclassification or reduced the accuracy of the outcome assessment. Nevertheless, this study shows a high statistical power as a large single-center retrospective analysis of 2,742 ACS patients who underwent coronary angiography and 2,323 individuals who underwent PCI strategy. To our knowledge, this is the first analysis specifically examining the association between Gensini scores and 30-day all-cause mortality among patients with ACS.

## Conclusions

In conclusion, among this large cohort of patients with ACS, higher Gensini scores were consistently associated with increased 30-day all-cause mortality across multiple subgroups, including sex, diabetes, hypertension, previous HF, previous MI, LM culprit, stent implantation, complete coronary revasculation, and ACS type. These findings underscore the value of the Gensini score as a simple and practical tool for early risk stratification. Further research is warranted to validate these results and explore potential interventions based on CAD severity assessed by Gensini scores to improve patient outcomes in this population.

## Supplementary Information

Supplementary Material 1.

## Data Availability

Not publicly available. Data are available from the authors upon reasonable request and with permission of the First Affiliated Hospital of Harbin Medical University and Qingpu Branch of Zhongshan Hospital Affiliated with Fudan University.

## Abbreviations

ACEI or ARB: angiotensin-converting enzyme inhibitors or angiotensin II receptor blockers
ACS: Acute Coronary Syndrome
CABG: Coronary Artery Bypass Graft
CAD: Coronary Artery Disease
CHD: Coronary Heart Disease
CK-MB: Creatine Kinase Myocardial Band Content
DBP: Systolic and Diastolic Blood Pressure
HF: Heart Failure
Hs-CRP: High-sensitivity C-reactive Protein Levels
HRs: Hazard Ratios
IABP: Intra-aortic Balloon Pump
LAD: Left Anterior Descending artery
LCX: Left Circumflex Artery
LVEF: Left Ventricular Ejection Fraction
LM: Left Main Coronary Artery
LMWH: Low Molecular Weight Heparin
MI: Myocardial Infarction
NSTE-ACS: non-ST-segment Elevation Acute Coronary Syndrome
NT-proBNP: N-terminal pro-brain natriuretic peptide
PCI: Percutaneous Coronary Intervention
RCA: Right Coronary Artery
ROC: Receiver Operating Characteristic
SBP: Systolic Blood Pressure
STEMI: ST-segment Elevation Myocardial Infarction
TnI: Troponin I
UFH: Unfractionated Heparin
