# Correction: The relationship between gensini score and rates of 30-day mortality in acute coronary syndrome patients in China

**DOI:** 10.1186/s12872-026-05821-w

**Published:** 2026-04-15

**Authors:** Yi Zhang, Rong Luo, Xiaochen Sun, Jiasheng Cai, Mohammed Ahmed Al-Kaif, Zhonghua Wang, Chunlei Li, Chuntao Wu, Zilong Wang, Zhuqin Li, Haibo Liu

**Affiliations:** 1 Departments of Cardiology, QingPu Branch of Zhongshan Hospital Affiliated to Fudan University, 1158 Park East Road, Shanghai, 201799 China; 2 Department of Cardiology, Huadong Hospiatal affiliated to Fudan University, 221 Yanan West Road, Shanghai, 200040 China; 3 Department of Cardiology, the First Affiliated Hospital of Harbin Medical University, 23 Youzheng Street, Harbin, 150001 China

**Correction: BMC Cardiovascular Disorders 26, 3 (2025)**

**https://doi.org/10.1186/s12872-025-05303-5**

In this article [1], the author’s name Mohammed Ahmed Al-Kaif was incorrectly written as Mohammed Ahmed Akkaif.

The original article has been corrected.
