# Supplementary material for: The relationship between Gensini score and rates of 30-day mortality in acute coronary syndrome patients in China
Source: BMC Cardiovasc Disord. 2025 Nov 29;26:3. doi: 10.1186/s12872-025-05303-5 (PMC12764064; doi:10.1186/s12872-025-05303-5)
Supplement: Supplementary file 1 — Supplementary Material 1. [file 12872_2025_5303_MOESM1_ESM.docx]

Supplemental Table 1. The principle of Gensini scoring system.

| Degree of coronary artery stenosis | Severity score | Coronary lesion site | Region multiplication factor |
| --- | --- | --- | --- |
| ≤ 25% | 1 | LM | 5 |
| 26% -50% | 2 | Proximal LAD or LCX | 2.5 |
| 51% -75% | 4 | Middle LAD | 1.5 |
| 76% -90% | 8 | Distal LAD | 1.0 |
| 91% -99% | 16 | Middle or distal LAD | 1.0 |
| 100% | 32 | RCA | 1.0 |
|  |  | Subbranch | 0.5 |

LM: left main coronary artery; LAD: left anterior descending; LCX: left circumflex coronary; RCA: right coronary artery.


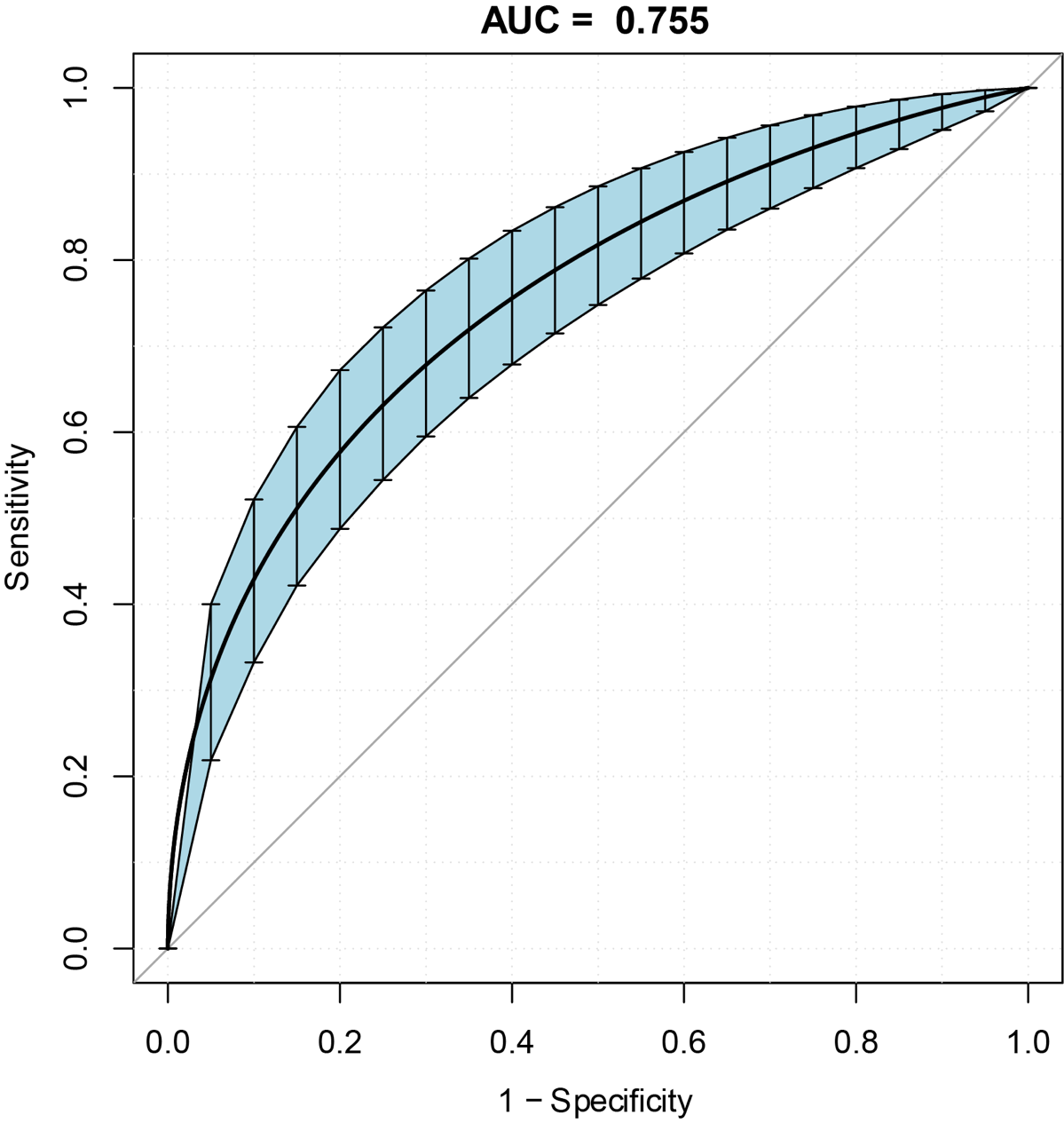
Supplemental Figure 1. Receiver operating characteristic (ROC) curves of the Gensini score for 30-day all-cause mortality.
